# TMPRSS2-ERG fusion promotes prostate cancer metastases in bone

**DOI:** 10.18632/oncotarget.14399

**Published:** 2016-12-31

**Authors:** Rachel Deplus, Carine Delliaux, Nathalie Marchand, Anne Flourens, Nathalie Vanpouille, Xavier Leroy, Yvan de Launoit, Martine Duterque-Coquillaud

**Affiliations:** ^1^ University Lille, CNRS, Institut Pasteur de Lille, UMR 8161 (M3T) Mechanisms of Tumorigenesis and Target Therapies, F-59000 Lille, France; ^2^ Institut de Pathologie Centre de Biologie Pathologie Centre Hospitalier Régional et Universitaire, F-59037 Lille, France

**Keywords:** TMPRSS2-ERG, prostate cancer, bone metastasis, bone tropism

## Abstract

Bone metastasis is the major deleterious event in prostate cancer (PCa). TMPRSS2-ERG fusion is one of the most common chromosomic rearrangements in PCa. However, its implication in bone metastasis development is still unclear. Since bone metastasis starts with the tropism of cancer cells to bone through specific migratory and invasive processes involving osteomimetic capabilities, it is crucial to better our understanding of the influence of TMPRSS2-ERG expression in the mechanisms underlying the bone tropism properties of PCa cells. We developed bioluminescent cell lines expressing the TMPRSS2-ERG fusion in order to assess its role in tumor growth and bone metastasis appearance in a mouse model. First, we showed that the TMPRSS2-ERG fusion increases cell migration and subcutaneous tumor size. Second, using intracardiac injection experiments in mice, we showed that the expression of TMPRSS2-ERG fusion increases the number of metastases in bone. Moreover, TMPRSS2-ERG affects the pattern of metastatic spread by increasing the incidence of tumors in hind limbs and spine, which are two of the most frequent sites of human PCa metastases. Finally, transcriptome analysis highlighted a series of genes regulated by the fusion and involved in the metastatic process. Altogether, our work indicates that TMPRSS2-ERG increases bone tropism of PCa cells and metastasis development.

## INTRODUCTION

Prostate cancer (PCa) is one of the most commonly diagnosed disease and the second cause of cancer-related deaths affecting men in Western world [1]. Bone metastases affect more than 80% of advanced stage PCa patients and constitute the major injurious events [2] with severe pain, nerve compression and pathologic fracture. PCa that has metastasized to bone remains incurable and more likely to lead to a fatal outcome than a primary tumor. Metastatic prostate cancer cells acquire a bone cell-like phenotype by a process called osteomimicry, which allows their survival and their proliferation in the bone marrow microenvironment [3, 4]. At this stage, the tightly-controlled balance between osteoblastic bone formation and osteoclastic bone resorption is aberrantly modified, thereby altering bone metabolism [5–7]. Contrary to osteolytic lesions observed in breast cancers, PCa bone metastases are mainly osteoblastic or mixed [8]. A major challenge for PCa treatment is to identify factors controlling tumor growth and metastases.

Gene fusions resulting from chromosomal rearrangements are known to play an important role in tumorigenesis and particularly in PCa [9, 10]. Tomlins *et al*. first described a gene fusion between the regulatory element of TMPRSS2 (Transmembrane Protease Serine 2) gene and ETS (*ERG, ETV1, ETV4* or *ETV5*) genes in PCa [11, 12]. TMPRSS2 is a prostate-specific androgen-regulated gene and its rearrangement leads to an increased in the expression of the ETS members in response to the androgen. ETS factors are a family of 27 transcription factors in mammals and sharing a conserved DNA binding domain recognizing 5’-GGAA/T-3’ sequence in promoters or regulation regions. ETS transcription factors play an important role in a variety of biological processes, including cell proliferation, apoptosis, differentiation, angiogenesis and invasiveness [13].

TMPRSS2-ERG gene fusions are one of the most predominant genetic events in PCa [10, 12]. More than 20 TMPRSS2-ERG transcripts were described as result of alternative splicing or different recombination mechanisms (deletions, insertions or translocations) [10, 14]. The most common rearrangement, involving exons 1 of TMPRSS2 fused to exon 4 of ERG (T1E4), is present in 50% of PCa cases [10]. Transcriptomic studies have revealed that ERG overexpression in TMPRSS2-ERG-positive PCa cell lines leads to the deregulation of key genes for cell migration and invasiveness [15–17].

The prognostic implications of TMPRSS2-ERG gene fusion in PCa are still unclear. Since its discovery in 2005, a large number of studies have been published in this field. Some teams found a correlation between the presence of this fusion and poor prognosis [18–22], but several others report a correlation between the presence of this fusion and good prognosis [23–25] or no correlation at all [26–28]. Moreover, several studies have demonstrated the presence of TMPRSS2-ERG gene fusion, in most cases of metastatic PCa [29, 30], and that positive tumors have a greater proclivity for developing metastases. On the other hand, metastases arising from tumors without rearrangement were also found [31]. This discrepancy could come from clinical settings, the size of the patients' cohort, the difference in sample collection or conservation, but also from PCa heterogeneity, fusion variant or ethnical differences.

Understanding the mechanisms of bone metastasis formation remains a challenge to improve PCa prognosis and treatment. This research field is a challenging area to explore because of the difficulty in obtaining bone metastatic tissues from patients. Animal models then become essential tools in exploring the pathogenesis of PCa. However very few cell lines reflect tumor colonisation, quiescence and subsequent growth in the bone following injection in the circulation [32].

In this paper, we used a luciferase expressing cell lines derivatives from PC3M, PC3M-luc-C6 (PC3M-luc), in a mouse model in order to evaluate the consequences of TMPRSS2-ERG fusion on tumor development using dynamic monitoring with bioluminescence imaging (BLI). New PC3M-luc TMPRSS2-ERG cells stably expressing the fusion showed increased migration ability and a higher subcutaneous tumor development. Moreover, we demonstrated that the presence of TMPRSS2-ERG results in a greater number of tumors in bone after intracardiac injection. In particular, site-specific patterns of spread are influenced by more lesions at hind limbs and spine for fusion positive cells. Finally, the TMPRSS2-ERG positive tumors harbour more osteoblastic features compared to TMPRSS2-ERG negative tumors. Our work provides new elements in the knowledge of bone metastasis development in PCa.

## RESULTS

### TMPRSS2-ERG fusion increases migration in prostate cancer cell lines

**Figure 1:**
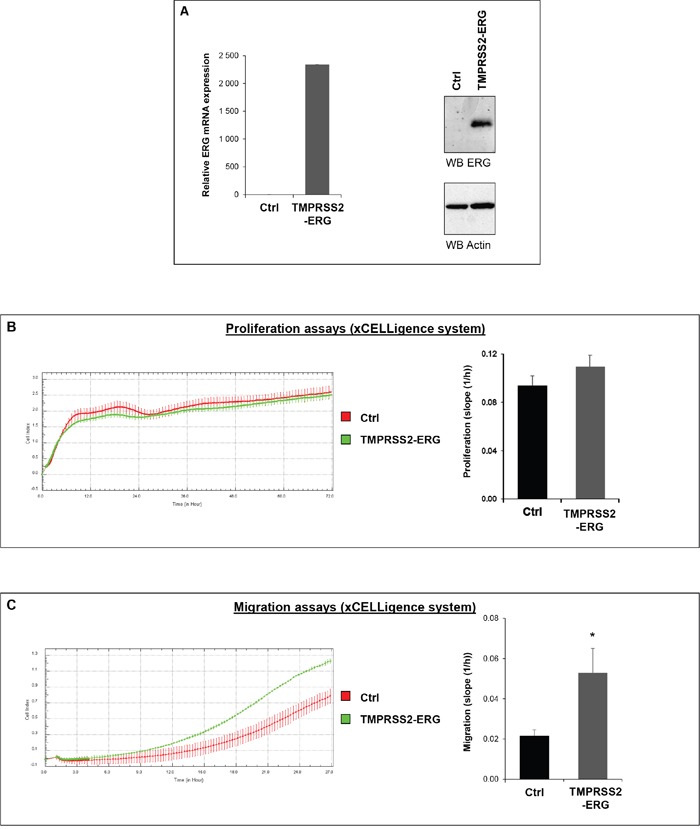
TMPRSS2-ERG fusion increases migration in prostate cancer cell line **A**. ERG expression in PC3M-luc cell lines. Left panel: ERG expression was evaluated by RT-qPCR analysis. Transcript levels were measured following infection of PC3M-luc with pLPCX empty vector (Ctrl) or pLPCX TMPRSS2-ERG (TMPRSS2-ERG). Results are normalized with respect to endogenous control GAPDH. Immunoblot analysis of ERG protein levels in PC3M-luc Ctrl or TMPRSS2-ERG. Actin was used as a loading control. Primer used are avalaible in Supplementary Table S1. **B**. Left panel: Representative cell index (mean ± standard deviation) as a measure of PC3M-luc cell proliferation. Measurements were automatically collected by the RTCA DP analyser every 15 min for up to 3 days (n=2). Red: PC3M-luc cells infected with pLPCX empty vector (Ctrl); Green: PC3M-luc cells infected with pLPCX TMPRSS2-ERG vector (TMPRSS2-ERG). Right panel: Results of the cell proliferation assays represented as slopes (changes in cell index/hour) (n=4) (*= p<0,05). **C**. Left panel: Migration kinetics PC3M-luc cells assessed by continuous monitoring of live cell migration for approximately 24 hours. Red: PC3M-luc cells infected with pLPCX empty vector (Ctrl); Green: PC3M-luc cells infected with pLPCX TMPRSS2-ERG vector (TMPRSS2-ERG) (n=2). Right panel: Results of the cell migration assays represented as slopes (changes in cell index/hour) (n=4) (*= p<0,05).

In order to assess the role of TMPRSS2-ERG fusion in PCa development, we generated cell lines stably overexpressing the fusion using retroviral translocation from luciferase expressing cells PC3M-luc. The expression of ERG was confirmed by RT-qPCR and immunoblotting (Figure 1A). Equivalent luciferase activity for both cell lines (PC3M-luc Ctrl and PC3M-luc TMPRSS2-ERG) was also checked to eliminate a possible bias in the further bioluminescence measurements (Supplementary Figure S1). Previously, it has been shown by our team and others that no significant cell proliferation changes were observed in TMPRSS2-ERG PC3c cells compared with control cells, but that TMPRSS2-ERG promotes PC3c cell migration [15–17]. To assess this influence in PC3M-luc cell line, we performed proliferation and migration assays in the xCELLigence system. This instrument monitors cellular events in real time without exogenous labelling through impedance-based technology [33]. Compared with control PC3M-luc cells (Ctrl), TMPRSS2-ERG overexpressing PC3M-luc cells exhibit comparable proliferation profiles (Figure 1B, right panel). The histograms presented in the left panel of Figure 1B show rates of estimated cell proliferation similar to the mean slope of the cell growth curves for four independent experiments. We then evaluated the effect of TMPRSS2-ERG on the migration behavior of PC3M-luc prostate cancer cells. As usual, we performed the migration assays using foetal bovine serum (FBS) as a chemoattractant. Cell migration was measured every 15 min for approximatively 24h. We found that the TMPRSS2-ERG cells migrate faster than the control cells (Figure 1C, left part). The rates of cell migration as the means of four independent experiments is presented in Figure 1C (right part). Our data confirmed previous observations [15–17] and thus demonstrate that TMPRSS2-ERG fusion also promotes cell migration in PC3M-luc.

### TMPRSS2-ERG fusion increases subcutaneous tumor growth

**Figure 2:**
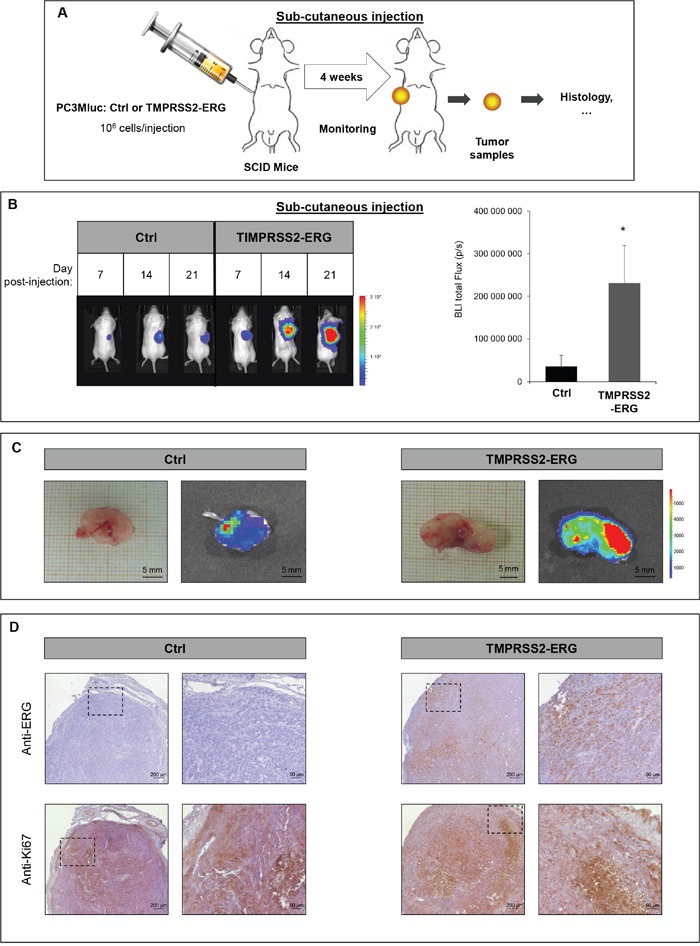
TMPRSS2-ERG fusion increases subcutaneous tumor growth **A**. Schematic representation of subcutaneous injection experiments. **B**. Bioluminescence imaging of SCID mice bearing PC3M-luc tumor cells (Ctrl or TMPRSS2-ERG). Left panel: Representative pictures on the indicated days after subcutaneous injections. Luminescence is expressed in radiance (p/sec/cm2/sr) and represented by the color scale. Right panel: Quantitative analysis of the luciferase expression as a measure of tumor growth at the end of the experiment (day 24). Data represents the means of 6 mice in Ctrl group and 5 mice in TMPRSS2-ERG group. * indicated p<0,05. **C**. Picture of excised (up) subcutaneous tumor at day 24 and bioluminescence (BLI, bottom) **D**. Histologic analysis of subcutaneous tumors with anti-ERG antibody (up) and anti-Ki67 antibody (bottom). Magnified insert are shown on the right.

The influence of TMPRSS2-ERG fusion on subcutaneous tumor growth has already been reported [14, 34, 35]. In an effort to characterize our new cell lines and to evaluate whether TMPRSS2-ERG could influence tumor development in an animal model system, we inoculated in PC3M-luc control or TMPRSS2-ERG cells the right flank of male SCID mice. Tumor development was monitored weekly by measuring bioluminescence with IVIS Lumina system (Figure 2A and Supplementary Figure S2). The growth rate was relatively rapid. Measurable tumors began to form 7 days after tumor cell inoculation in all the injected mice. At the end of the experiment, the presence of TMPRSS2-ERG fusion resulted in a luciferase signal six-fold higher than Ctrl cells (Figure 2B). Representative time course of tumor development in mice injected with Ctrl cells or TMPRSS2-ERG cells is presented in Figure 2B and Supplementary Figure S2. As can be seen from Figure 2C, subcutaneous tumors formed by TMPRSS2-ERG cells were much more bioluminescent than those formed by Ctrl cells. Confirmation of the TMPRSS2-ERG status was obtained on the tumor sample by immunohistochemistry with anti-ERG (Figure 2D). Using an anti-Ki67 antibody, we validated the high proliferation rate in both tumor groups (Figure 2D).

### TMPRSS2-ERG fusion increases dissemination in bone mainly in hind limbs and the spine

**Figure 3:**
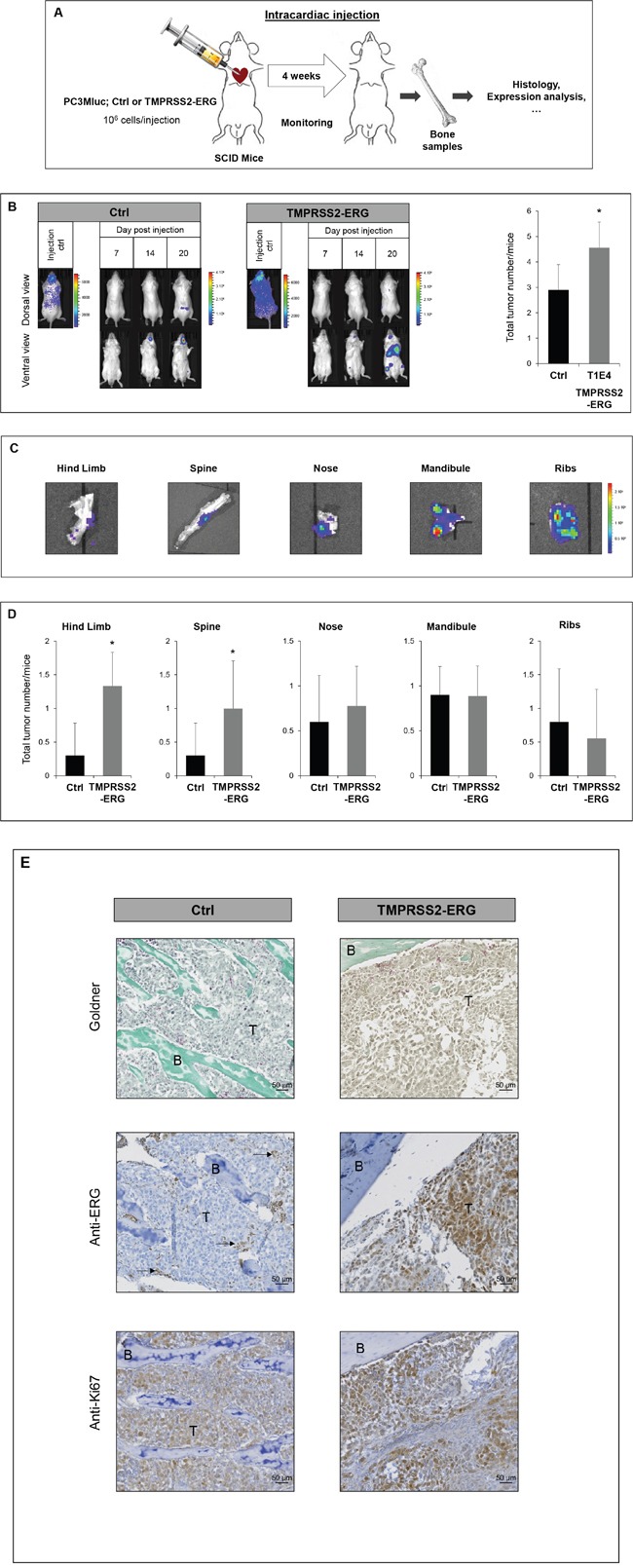
TMPRSS2-ERG fusion increases bone metastasis development **A**. Schematic representation of intracardiac injection experiments. **B**. Bioluminescence imaging of SCID mice bearing PC3M-luc tumor cells (Ctrl or TMPRSS2-ERG). Left panel: Representative bioluminescence imaging of SCID mice on the indicated times after intracardiac injections. Right panel: Number of tumors per mice at indicated days after intracardiac injections. Data represents the mean of 10 mice in Ctrl group and 9 mice in TMPRSS2-ERG group. Luminescence is expressed in radiance (p/sec/cm2/sr) and represented by the color scale. * indicated p<0,05. **C**. *Ex vivo* detection of bone tumor by bioluminescence at day 24. Luminescence is expressed in radiance (p/sec/cm2/sr) and represented by the color scale. **D**. Number of tumors per mice in indicated organ sites at day 24 after intracardiac injections. Data represents the mean of 10 mice in Ctrl group and 9 mice in TMPRSS2-ERG group. * indicated p<0,05. **E**. Histologic analysis of bone tumors resulting from intracardiac injection of PC3M-luc Ctrl (left) or PC3M-luc TMPRSS2-ERG (right) by Goldner staining (top), with anti-ERG antibody (middle) and anti-Ki67 antibody (bottom). B=Bone, T= Tumor cells. Arrows indicate ERG positive vessels cells.

Given that TMPRSS2-ERG increases both the migration *in vitro* and the subcutaneous tumor development, we then tested whether TMPRSS2-ERG could be taking part in the bone metastasis formation of prostate cancer cells *in vivo*. In this view, we used an experimental metastasis model involving intracardiac injections of PC3Mluc tumor cells in SCID mice. This approach mimics the haematogenous dissemination of cancer cells and allows the examination of the process of metastatic colonization at various sites. PC3M-luc Ctrl and TMPRSS2-ERG were injected in the left ventricle of male SCID mice aged of six weeks (Figure 3A and Supplementary Figure S3). The distribution of the cells in the whole body 30 minutes after injection, indicating successful injection, was apparent for 65% of the mice. Only animals with diffuse photon accumulations throughout the body were considered for further analysis. Then, images were taken twice per week to monitor the cell colonization. Mice were euthanized at day 24. These experiments were repeated twice and similar results were obtained. In total, 10 mice were used to inject PC3M-luc Ctrl cells and 9 mice with PC3M-luc TMPRSS2-ERG.

Tumor cell dissemination was rapidly detectable. Firstly, we observed a colonization of the nose and mandibles (from day 4, with high sensitivity detection). Luciferase was later detected in hind limbs and the spine (around day 14) (Figure 3B and Supplementary Figure S3). 24 days after injection 100% of both groups of mice had developed bone metastatic foci and whole body luminescent was comparable between the two groups (Supplementary Figure S3B). Nevertheless, the fusion status influences the total number of metastatic sites. Animals injected with TMPRSS2-ERG cells had 57% more bone metastases compared to the control group (Figure 3B) at the end of the experiment.

A more detailed examination revealed that TMPRSS2-ERG leads to a higher number of tumors in hind limbs and the spine (Figure 3C, 3D). Other sites, such as the mandible, nose or ribs were colonized in a similar way by both cell lines. Supplementary Figure S4 shows the incidence of tumoral lesions per site for each mouse considered in our experiment. Macroscopic dissection and bioluminescence measurement analysis of metastatic sites confirmed luciferase detection corresponding to the presence of tumor cells (Figure 3C). Goldner coloration of the collected bone samples confirmed bone tumor localisation. A histological analysis with antibodies against ERG and Ki67 respectively confirmed the TMPRSS2-ERG status and the high rate of proliferation (Figure 3E and Supplementary Figure S5). As expected, the endothelial cells of small vessels show positive endogenous ERG and are indicated with arrows.

Altogether, these observations show that the fusion TMPRSS2-ERG plays an important role tumor cell dissemination into the bone, by increasing the incidence of bone metastases in hind limbs, in the spine and globally.

Although the clinical PCa bone metastases have the predominant osteoblastic phenotype, PCa cell lines induced mainly osteolytic lesions [36]. In order to characterize bone metastases, we compared the transcript levels of some osteoblast-specific markers (OSTERIX and RUNX2) and osteoclast-related markers (RANK) in PC3M-luc Ctrl or PC3M-luc TMPRSS2-ERG. Supplementary Figure S6A shows the expression levels in the spine sample of three different mice to account for inter-individual variability. These results suggest a deregulation of the bone metabolism in favour of an osteoblastic phenotype as indicated by the trend towards an increase in the expression of osteoblastic markers (OSTERIX and RUNX2) and a decrease in the expression of the osteoclastic key gene (RANK) (Supplementary Figure S6A). Samples were also assessed by histological analysis. In samples from mice injected with TMPRSS2-ERG cells, we noticed an increase in bone matrix as highlighted in Supplementary Figure S6B with arrows. Finally, we performed immunohistochemistry with an anti-RUNX2, which revealed more positive cells in bone samples from mice injected with TMPRSS2-ERG cells compared with Ctrl cells. To complete this histological study, we performed immunohistochemistry experiments on mouse bone lesions to detect the Cathepsin K expression, which is specific to osteoclasts. As shown in Supplementary Figure S6C, Cathepsin K was detected in both Ctrl and TMPRSS2:ERG bone lesions in a similar manner. This result suggests that, even though the fusion expression is associated with osteoblastic feature as revealed by the Goldner staining and the osteogenic Runx2 gene expression, the TMPRSS2:ERG expression does not decrease the osteoclasts number in this model.

### TMPRSS2-ERG fusion expression deregulates genes involved in cell migration, adhesion and skeletal physiology

**Figure 4:**
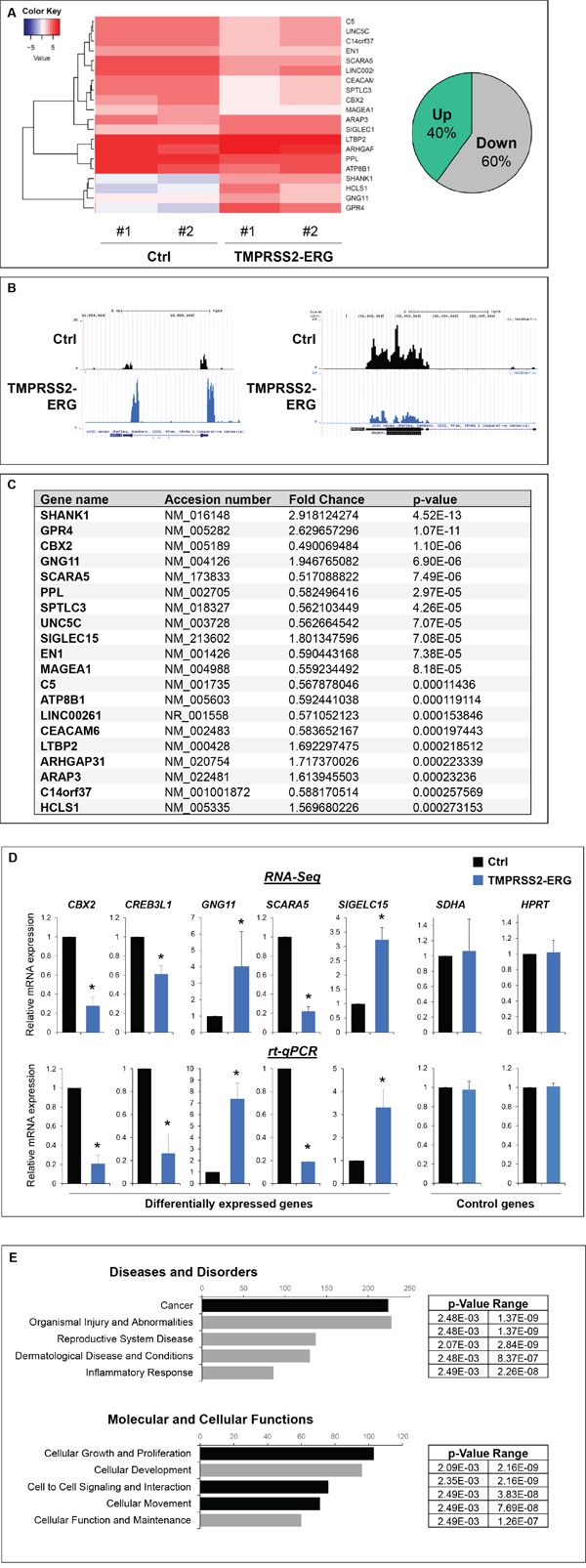
TMPRSS2-ERG fusion expression deregulates genes involved in cell migration, adhesion and skeletal physiology **A**. RNA-sequencing analysis. Left panel, Heatmap showing top 20 of differentially expressed genes between PC3M-luc Ctrl and TMPRSS2-ERG cells. A technical duplicate was analysed for each sample (#1 and #2). The heatmap scale shows the range of expression level, whereby positive (red) and negative (blue) values correspond, respectively, to a higher and a lower expression. Right panel, pie chart showing the percentage of significatively (p value <0.05) up and downregulated genes in TMPRSS2-ERG cells versus control cells. **B**. Examples of RNA-Seq peaks (UCSC traks). **C**. Top 20 of significatively deregulated genes. **D**. Validation of expression changes observed in RNA-Seq by q-PCR. Results are normalized with respect to endogenous control GAPDH. **E**. Ingenuity pathway analysis (“Diseases and Disorders” and “Molecular and Cellular Function”) of the differentially expressed genes. The x axis represents the number of molecules per categories.

Since TMPRSS2-ERG fusion product correspond to a functional transcription factor, we speculated that its over-expression in PC3M-luc cells, induces differences in their transcriptome. To address the biological functions of TMPRSS2-ERG in bone metastasis formation, RNA sequencing was employed to compare the global gene expression of PC3M-luc TMPRSS2-ERG cells versus Ctrl cells (Figure 4, Supplementary Figure S7 and Supplementary Table S2-S6). Overall, the quality of the RNA-Seq data and read mapping in our current study met the requirements for the bioinformatic analyses. Moreover, the expression levels of a set of genes in the corresponding clones were validated using qRT-PCR (Figure 4E). These data confirmed the RNA-seq results and showed the significantly up- and down-expression of the genes. In order to explore the gene function relevant to the *in vitro* and *in vivo* results obtained with the PC3M-luc TMPRSS2-ERG clones, GO analysis was conducted to group these differentially expressed genes into molecular and cellular functions, diseases and physiological system development and function (Figure 4E and supplementary Figure S7). In brief, GO analysis of genes showed that these genes are involved in cellular growth and proliferation, cell movement, tissue development, skeletal and muscular system development (Figure 4 and Supplementary Figure S7 and Table S2-5). Most of the identified genes are involved in cancer and metastasis (Supplementary Table S2). More importantly, a large part of the deregulated genes are in the GO category “Connective tissues” (Supplementary Table S5) and involved in “Differentiation of bone cells” such as CCL2, INHBA, ITGA5, NOS3, SIGLEC15, WNT7B or in “Development of connective tissue” such as CXCL11, ICAM1, INHBA, ITGA5, ITGB8, MMP13, PECAM1, PLA2G4A, WNT7A, WNT7B. Interestingly, some genes belong to the “Homing of cell” GO categories (20 genes), others to “Migration of cells” (60 genes) or to the “Extravasation” (6 genes) which is an important step of metastatic mechanism (Supplementary Table S3 and S4). In summary, over-expression of TMPRSS2-ERG was sufficient to modulate transcription of genes involved in cell migration/adhesion and mechanisms known to be associated with bone physiology. In particular, de-regulation of mRNAs whose gene products are involved in cancer and metastasis was observed.

## DISCUSSION

PCa is a global public health problem, and in particular bone metastasis development which is responsible for main morbidity. Bone lesions are difficult to cure and are often synonym of fatal outcome. Therefore, there is an urgent need to develop therapeutic strategies that target advanced PCa and its interactions with the bone. A prerequisite for the development of new therapeuties is to improve our understanding of the fundamental mechanisms that regulate the metastatic process, including dormancy and growth of tumor cells in the bone. Previous studies failed to reach clear conclusions about the role of TMPRSS2-ERG in bone metastases. Finding good and relevant models is a real challenge in prostate cancer research. In this view, we developed PC3M-luc cells lines stably overexpressing the fusion TMPRSS2-ERG.

In this study, *in vitro* experiments showed that the TMPRSS2-ERG fusion increases cell migration. This result is in agreement with previous studies obtained with PC3 and other cell lines [15–17], confirming the validity of our PC3M-luc model. By way of subcutaneous injection, we clearly demonstrated that TMPRSS2-ERG overexpression leads to higher bioluminescent signal, which reflects the presence of more tumor cells. This last data, in addition to be in line with *in vitro* migration results, suggests that the fusion may be involved in microenvironment interactions enhancing tumor growth *in vivo*. Several previous studies have demonstrated the presence of the TMPRSS2-ERG gene fusion in the majority of metastatic PCa [29, 30, 37] and that the positive foci have a greater proclivity for metastases [29]. Nevertheless, others reported that the metastases may also arise from the tumor without ERG rearrangement [31]. Animal models are very useful tools to better understand mechanisms involved in PCa metastases. Here, we used intracardiac injections to evaluate whether the TMPRSS2-ERG fusion plays a role in tumor propagation to the bone. This technology imitates the propagation of cancer cells in blood circulation and is the method of choice to follow the homing of PCa cells in various tissues. Our work showed that PC3M-luc cells, expressing TMPRSS2-ERG, show a higher rate of colonization in bone compared to Ctrl cells. Crucially, this indicates a major involvement of the fusion in bone metastases, and therefore suggests that the TMPRSS2-ERG fusion may play a role in bone tropism of PCa tumor cells.

Interestingly, anatomical sites presenting increased tumors are hind limbs and spine, which are two of the most affected tissues in human pathology [38, 39]. Metastatic PCa cells in the spine and hind limbs provide some of the worst symptoms of the disorder due to pathological fracture or epidural spinal cord compression [40]. The fact that these two bone lesion localisations reflect human pathologies demonstrates that the intracardiac injection of PC3M-luc cells are a good and relevant model to study PCa pathology.

It was suggested that osteotropism of PCa cells is a consequence of the development of a bone-like behavior. This theory, called osteomimicry, is the ability of cancer cells to express genes previously highly restricted to bone cells during and after metastasis in order to adapt and grow in the bone environment [3, 4, 41, 42]. We have shown here that TMPRSS2-ERG influences the transcription of bone master genes, suggesting that the fusion TMPRSS2-ERG may be a driver of osteomimicry. Moreover, TMPRSS2-ERG seems to destabilize the bone metabolism by increasing the transcript level of two osteoblastic genes, being RUNX2 and OSTERIX [43, 44], and decreasing the expression of an osteoclastic gene, RANK [45]. However, using immunohistochemistry experiments on mouse bone lesions to detect Cathepsin K expression, which is specific to osteoclasts, we revealed that, compared to Ctrl tissue, osteoclast number was not decresed in TMPRSS2-ERG bone lesions. This result suggests that, even though the fusion expression is associated with osteoblastic feature as revealed by the Goldner staining and the osteogenic RUNX2 gene expression (Supplementary Figure 6A-6B), the TMPRSS2- ERG expression does not decrease the osteoclast number in this model. In line with this results, Li *et al*. showed that osteoclastogenesis and bone resorption are mutually essential for prostate cancer establishment in the bone microenvironment [46]. Moreover, they demonstrated that CCL2 (Monocyte chemoattractant protein 1) increased osteoclastic bone resorption to facilitate prostate cancer tumor growth in bone. Interestingly, as revealed by RNA-Seq analysis, CCL2 gene is upregulated in PC3M-luc TMPRSS2-ERG clone compared with the Ctrl clones. In this respect, the TMPRSS2-ERG fusion seems to favour the development of osteoblastic lesions. Since ERG is a transcriptional factor which could act as an activator of transcription but also as a transcriptional repressor [47, 48], the effect of TMPRSS2-ERG on RUNX2, OSTERIX, RANK and CCL2 expression, suggests that these genes could be potential ERG targets. Further studies should be performed to establish if these genes are directly bound by ERG factor.

Our results suggest that the TMPRSS2-ERG fusion may be a target to inhibit bone metastases. Further to our results, it would be interesting to assess if treatment with ERG inhibitors could reduce the number of developed bone metastases. An inhibitor such as DB1255, which specifically targets the ERG DNA recognition sites [49] or ERG-specific siRNA [34] could be potential anti-metastatic compounds. Positive results could open new promising therapeutic possibilities to treat prostate cancer bone metastases.

In summary, our findings show that the TMPRSS2-ERG fusion play a role in PCa bone metastasis formation.

## MATERIALS AND METHODS

### Cell lines and retroviral infection

PC3M-luc-C6 cell line was purchased from Caliper (Perkin Elmer). PC3M-luc are derived from PC3 cell line, originally isolated from a bone metastasis of human prostatic adenocarcinoma [50] [51]. To produce highly metastatic cells, they were obtained after multiple selection cycles of orthotopic injection, after the subsequent isolation of metastatic cells and after orthotopic reinjection(s) [52].

Cells were grown in MEM (Life Technologies) medium containing 10% foetal bovine serum and glutamine (2mM final) under standard culture conditions. Retroviral infections to stably expressed the TMPRSS2-ERG fusion were realized as already described [15]. Briefly, 293 GP cells were transfected with pLPCX retroviral vectors. Supernatant were then collected and used to infect PC3M-luc-C6 target cells. For further experiments, we used a pool of several clones obtained by puromycine selection without clonal selection.

### Cell proliferation and migration assays

To evaluate PCa cell proliferation, PC3M-luc-C6 cells infected with pLPCX empty vector (Ctrl) or pLPCX TMPRSS2-ERG were seeded into the xCELLigence E-plate 16 (Roche) (10000 cells/well) according to the manufacturer's instructions. Measurements were collected by the RTCA DP analyser for up to 3 days. Four replicate measurements per condition were obtained. The data were analysed with the provided RTCA software. To examine PCa cell migration, PC3M-luc-C6 cells Ctrl or TMPRSS2-ERG were seeded 24 h post-transfection into the xCELLigence CIM-plate 16 (Roche). Briefly, a 165-μl volume of fresh medium containing 10% FBS (chemoattractant) or with serum-free medium (control) was added to the lower chambers of the CIM-plate 16. The upper chambers were filled with serum-free medium (30 μl/well) and the plate was incubated at 37°C in 5% CO2 for 1 h. Cells (60000 cells/well) were then added to each well of the upper chamber. After 30 min, the CIM plate was assembled onto the RTCA DP analyser and cell migration was assessed at 20 h at 37°C in 5% CO2. Four replicate measurements per condition were obtained. The data were analysed with the provided RTCA software.

### Animal tumor models

All animal experiments were approved by the local ethics committee (CSTMT-042). Six to eight week old male SCID mice were used. PC3M-luc-C6 (3.10^6^) suspended in 200μl of PBS were implanted subcutaneously in the flank region of SCID mice. For intracardiac injections, mice were maintained under isoflurane anaesthesia during injection procedure. PC3M-luc-C6 (10^6^) were suspended in 200μl of PBS and were injected into the left ventricle using a29-G needle. Bioluminescence imaging was performed 30 minutes after intracardiac injection. Only animals with diffuse photon accumulations throughout the body of the animal were considered for further analysis. Animals were monitored for 4 weeks after injection.

### Bioluminescence imaging

Mice were injected subcutaneously with D-luciferin (15 mg/ml in sterile PBS, Perkin Elmer) 10 min before being imaged. The mice were anesthetized with 2% isoflurane and were imaged in dorsal and/or ventral position using an IVIS Lumina System (Caliper) to monitor the tumor growth and metastatic dissemination. A bioluminescent image was obtained with a 12.5 cm field of view, a binning factor of 8, and a 1/f stop-and-open filter. Regions of interest were defined manually, and signal intensities were calculated with Living Image software (Caliper) and expressed as photons per second. Background photon flux was defined from a region of interest drawn over a control.

### RNA-sequencing

The total RNA was purified by RNeasy kit (Qiagen) from two distinct infections (#1 and #2) of PC3M-luc ctrl and TMPRSS2-ERG was used to perform RNA sequencing. Construction of the library was done with 1μg of total RNA and Tru-seq stranded mRNA sample preparation kit (Illumina) according manufacturer's instructions. The reads were aligned to the hg19 reference assembly using RNA-STAR [53]. Counting reads were mapped to genes with HTSeq-count under the union-intersection mode on refSeq hg19 transcriptome annotations. PCR duplicates were filtered. A gene was considered differentially expressed at transcript level when unpaired t-test P-value <0.05.

### Statistical analysis

All data are expressed as mean ± SEM. Statistical significance was tested for using an unpaired Student's t-test. p < 0.05 was considered to be significant and is indicated as *.

## SUPPLEMENTARY MATERIALS FIGURES AND TABLES












