# Supplementary material for: TMPRSS2-ERG fusion promotes prostate cancer metastases in bone
Source: Oncotarget. 2016 Dec 31;8(7):11827–40. doi: 10.18632/oncotarget.14399 (PMC5355307; doi:10.18632/oncotarget.14399)
Supplement: Supplementary file 1 [file oncotarget-08-11827-s001.pdf]

TMPRSS2-ERG fusion promotes prostate cancer metastases in bone

SUPPLEMENTARY MATERIALS AND METHODS

RNA purification and quantitative real-time PCR assays

Extraction of total RNA was carried out with the TriPure reagent (Roche) according to the manufacturer's instructions. DNase treatment was performed with a DNA-free DNase kit (Ambion) according to the manufacturer's protocol. Quantitative PCRs were performed using SYBR Green dye (Eurogentec) on a LightCycler 480 (Roche). Briefly, cDNA was reverse transcribed from 1 µg of RNA using random hexamers (Invitrogen) and Superscript II reverse transcriptase (Invitrogen). To quantitatively evaluate gene expression levels, real-time PCRs were performed. The primer sequences are described in Supplementary Table 1. GAPDH was amplified as an internal control. In Figure 4A, total RNA was extracted for spine tumors of mice injected in left ventricle with PC3M-luc ctrl or TMPRSS2-ERG cells. Primer used are specific of human template. Data are shown as mean of duplicates for each mice.

Cell extracts, immunoprecipitations and western blot analyses

Whole-cell extracts were prepared using IPH lysis buffer (50 mM Tris-HCl pH 8, 150 mM NaCl, 5mM EDTA, 0.5% NP40). All procedures were performed at 4°C. Standard procedures were used for Western blotting [15]. Primary antibodies used in these experiments were anti-ERG (ab92513, Abcam) and anti-Actin (A5316, Sigma).

Cell proliferation and migration assays

To evaluate PCa cell proliferation, PC3M-luc-C6 cells infected with pLPCX empty vector (Ctrl) or pLPCX TMPRSS2-ERG were seeded into the xCELLigence E-plate 16 (Roche) (10000 cells/well) according to the manufacturer's instructions. Measurements were collected by the RTCA DP analyser for up to 3 days. Four replicate measurements per condition were obtained. The data were analysed with the provided RTCA software. To examine PCa cell migration, PC3M-luc-C6 cells Ctrl or TMPRSS2-ERG were seeded 24 h post-transfection into the xCELLigence CIM-plate 16 (Roche). Briefly, a 165-µl volume of fresh medium containing 10% FBS (chemoattractant) or with serum-free medium (control) was added to the lower chambers of the CIM-plate 16. The upper chambers were filled with serum-free medium (30 µl/well) and the plate was incubated at 37°C in 5% CO2 for 1 h. Cells (60000 cells/well) were then added to each well of the upper chamber. After 30 min, the CIM plate was assembled onto the RTCA DP analyser and cell migration was assessed at 20 h at 37°C in 5% CO2. Four replicate measurements per condition were obtained. The data were analysed with the provided RTCA software.

Staining and immunohistochemistry

After imaging, the samples were fixed with paraformaldehyde (4%). Bones were decalcified with Osteosoft (Merk) according manufacturer's recommendations. All sample were embedded in paraffin after tissue processing (dehydration, clearance and impregnation). Serial 7-µm paraffin sections were processed and routinely stained with Goldner trichrome. For immunohistochemistry, anti-ERG (ab92513, Abcam) was used at a concentration 1/100, anti-Ki67 (ab15580, Abcam) at 1/200, anti-Runx2 (sc 10758, Santa Cruz) at 1/100.

SUPPLEMENTARY FIGURES AND TABLES

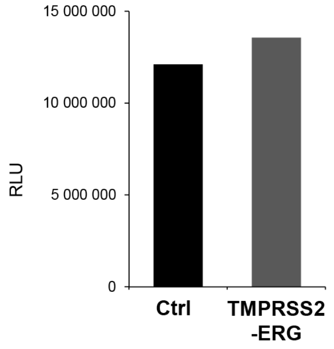

Supplementary Figure 1 (related to Figure 1): Luciferase activity for PC3M-luc Ctrl and PC3M-luc TMPRSS2-ERG. Representative data are shown.

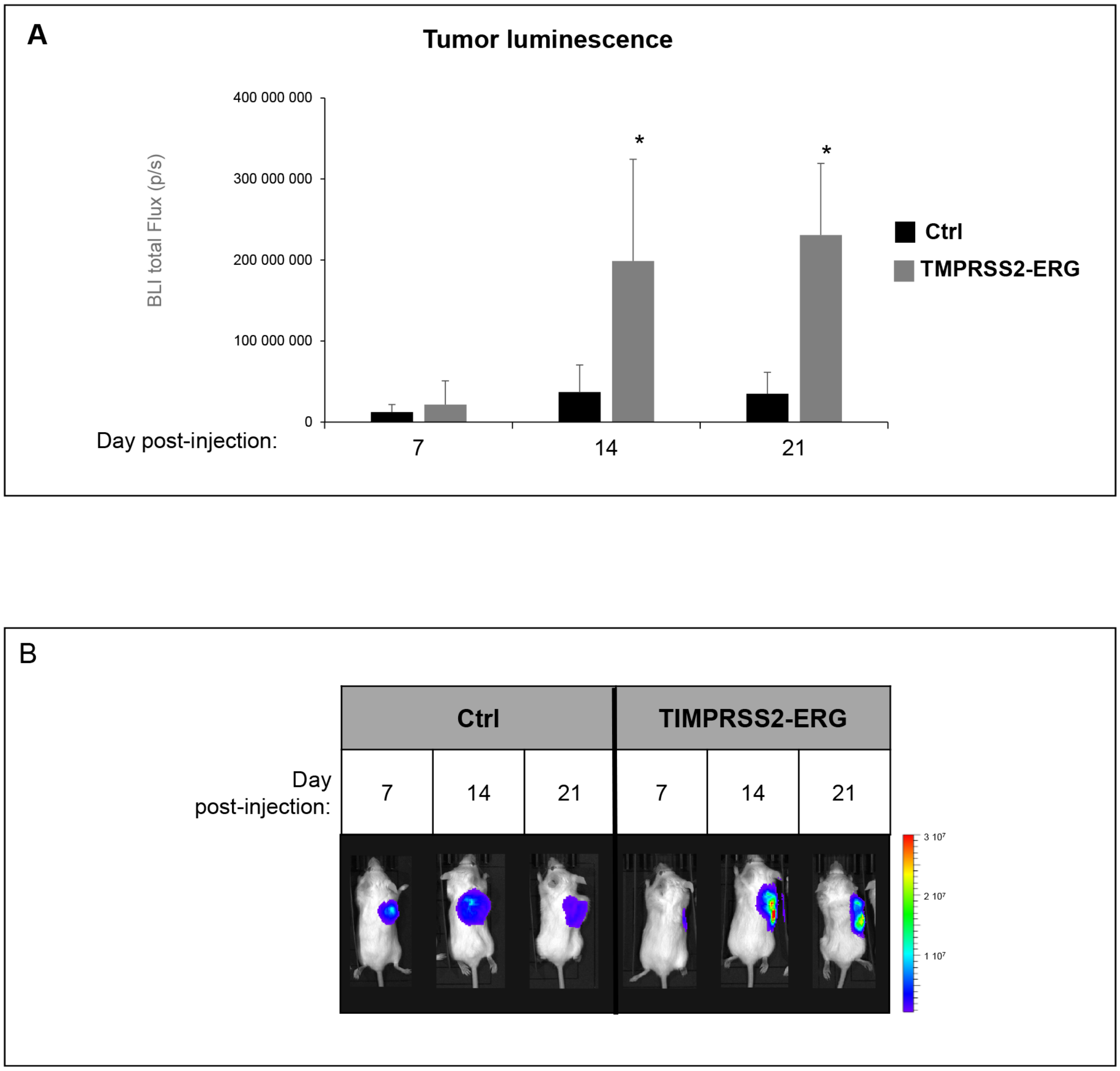

Supplementary Figure 2 (related to Figure 2): A. Bioluminescence imaging of SCID mice bearing PC3M-luc tumor cells (Ctrl or TMPRSS2-ERG). Quantitative analysis of the luciferase expression as a measure of tumor growth at indicated time. Data represents the means of 6 mice in Ctrl group and 5 mice in TMPRSS2-ERG group. * indicated p<0,05. **B.** Additional exemple of bioluminescence imaging of SCID mice bearing PC3M-luc cells (Ctrl or TMPRSS2-ERG). Luminescence is expressed in radiance (p/sec/cm2/sr) and represented by the color scale.

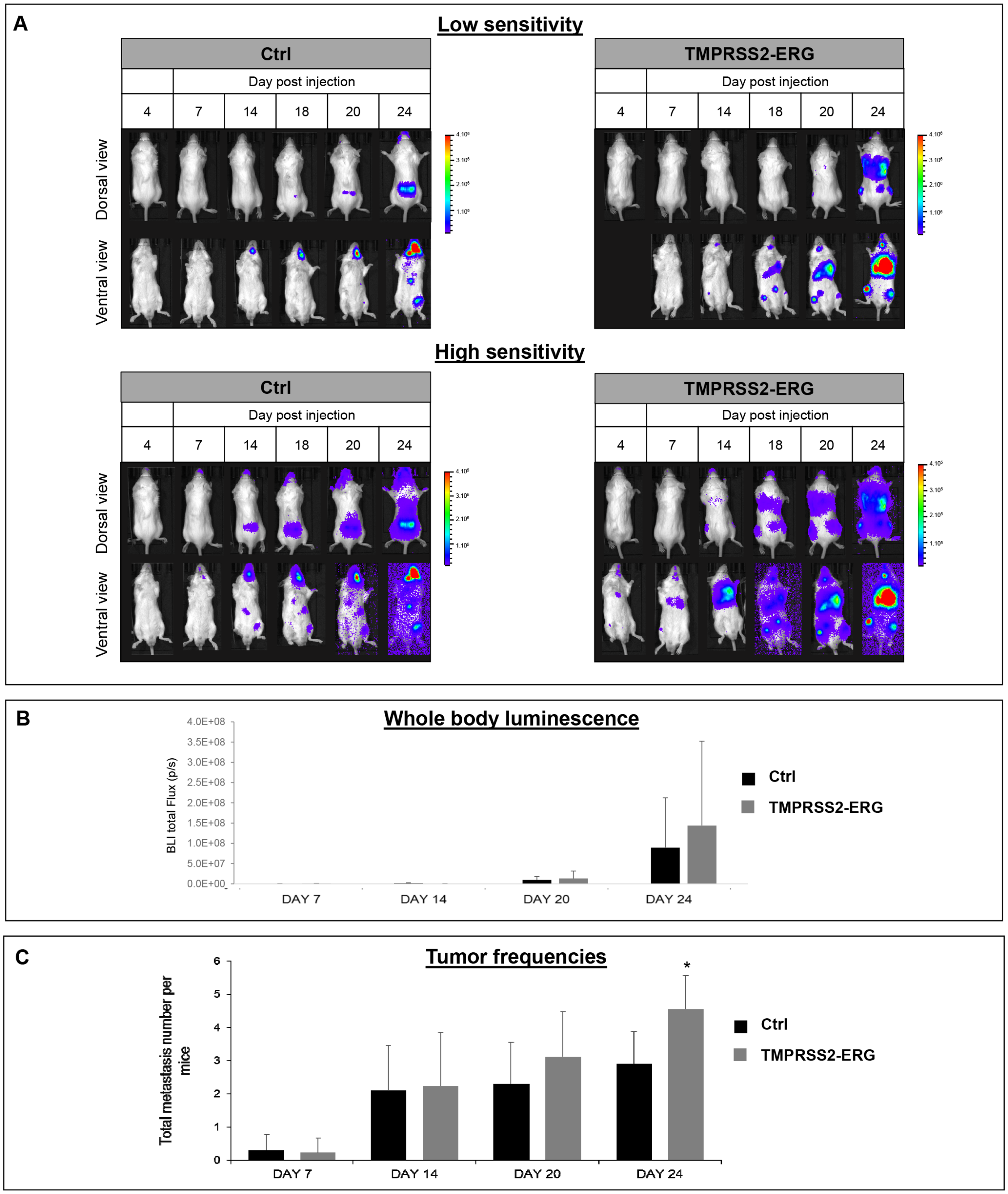

Supplementary Figure 3 (related to Figure 3B): A. Representative bioluminescence imaging of SCID mice bearing PC3M-luc tumor cells (Ctrl or TMPRSS2-ERG) at the indicated times after intracardiac injections with low or high sensitivity detection parameters (top and bottom, respectively). Luminescence is expressed in radiance (p/sec/cm2/sr) and represented by the color scale. **B.** Whole body luminescence of mice after intracardiac injections at indicated time. **C.** Number of tumors per mice at indicated days after intracardiac injections. Data represents the mean of 10 mice in Ctrl group and 9 mice in TMPRSS2-ERG group. * indicated p<0,05.

A

Cell line	Mouse	Nose	Mandibule	Himd Limb	Spine	Ribs	Total/ mouse
Ctrl	1	1	1			1	3
	2	1	1	1		1	4
	3	1					1
	4	1	1		1		3
	5		1			2	3
	6		1	1	1		3
	7		1			1	2
	8		1	1	1	1	4
	9	1	1				2
	10	1	1			2	4
	Total/ site	6	9	3	3	8	29
TMPRSS2-ERG	1	1	1	1	2		5
	2	1	1	2	1	1	6
	3	1	1	1	1		4
	4	1	1	1	1		4
	5	1	1	2			4
	6	1	1	1		1	4
	7	1		2	1	2	6
	8		1	1	1		3
	9		1	1	2	1	5
	Total/ site	7	8	12	9	5	41

B

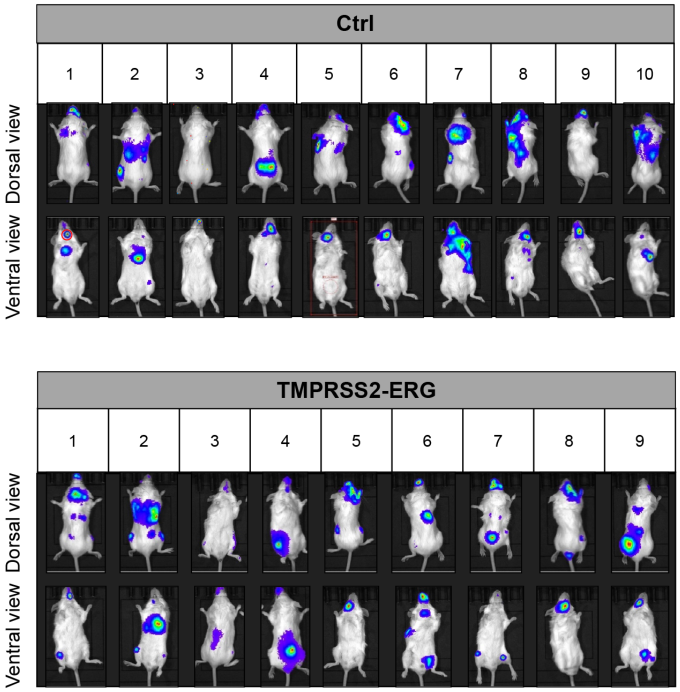

Supplementary Figure 4 (related to Figure 3): A. Incidence of metastasis to organ sites for mice injected in left ventricle with PC3M-luc Ctrl or TMPRSS2-ERG at the end of experiment. **B.** Bioluminescence imaging of all SCID mice bearing PC3M-luc tumor cells (Ctrl or TMPRSS2-ERG) after intracardiac injection. Automatic parameters were used to detected all tumors. Macroscopic dissection and bioluminescence measurement analysis of metastatic sites confirmed luciferase detection corresponding to the presence of tumor cells (Figure 3C).

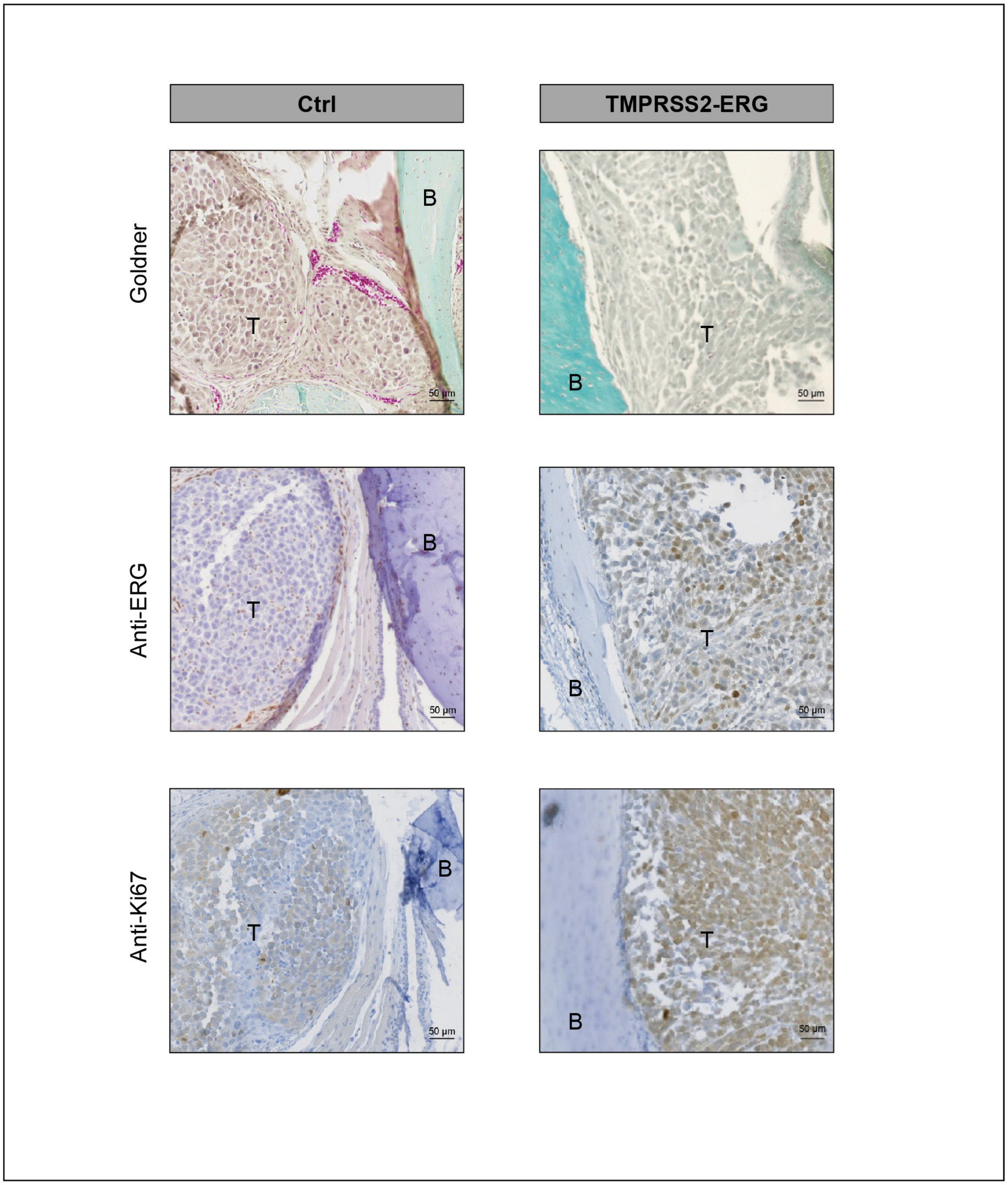

Supplementary Figure 5 (related to Figure 3E): Additional Histologic analysis of bone tumors resulting from intracardiac injection of PC3M-luc Ctrl (left) or PC3M-luc TMPRSS2-ERG (rigth) by Goldner staining (top), with anti-ERG antibody (middle) and anti-Ki67 antibody (bottom). B=Bone, T= Tumor cells.

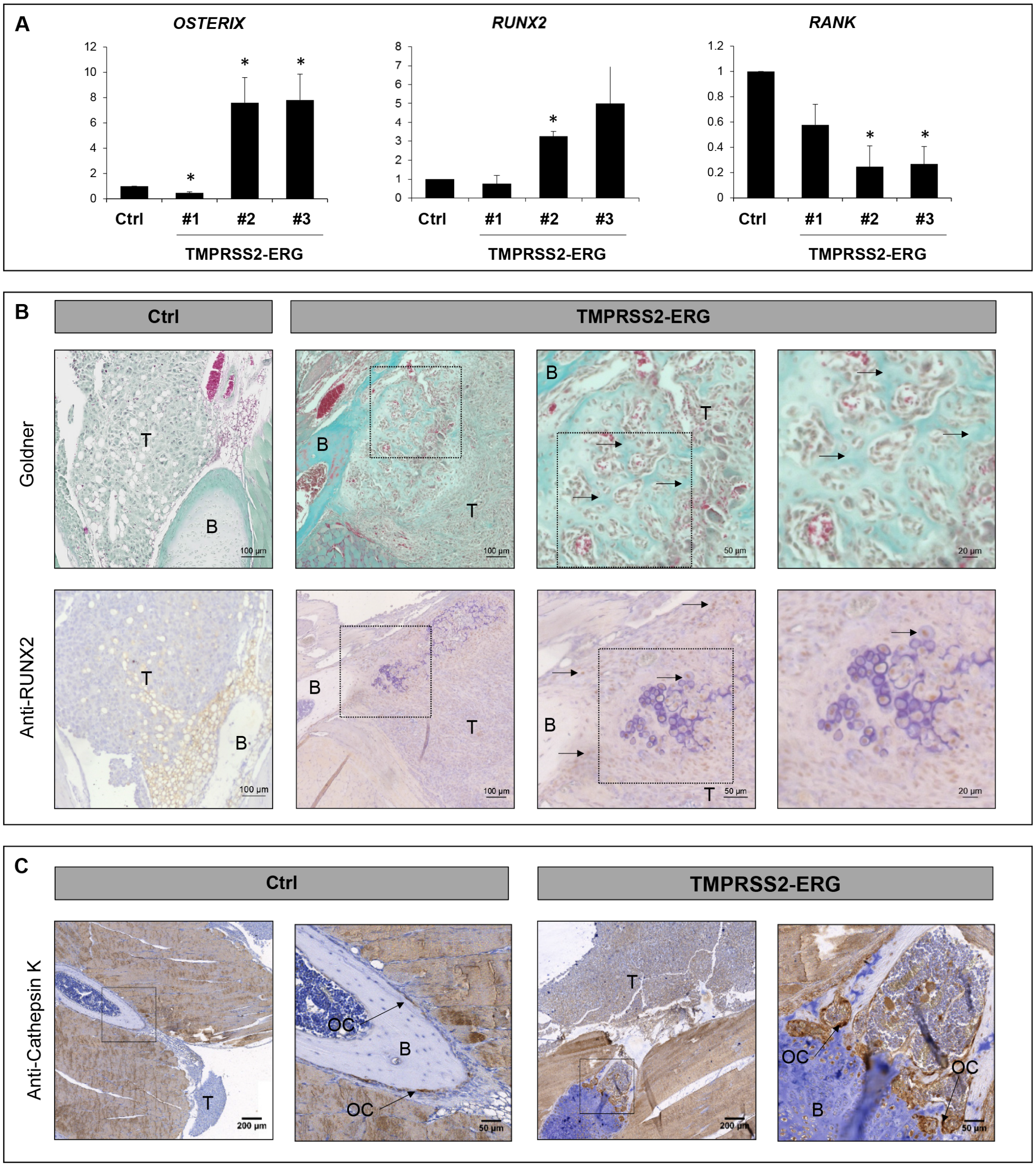

Supplementary Figure 6 (related to Figure 3): A. Real-Time PCR analysis of *Osterix*, *Runx2* and *RANK* in spine of mice injected in left ventricle with PC3M-luc Ctrl or TMPRSS2-ERG cells. Data are shown as mean of duplicates for each mice. Results are normalized with respect to endogenous control GAPDH. (*= p<0,05). **B-C.** Histologic analysis of bone tumors resulting from intracardiac injection of PC3M-luc Ctrl (left) or PC3M-luc TMPRSS2-ERG (right) by Goldner staining (B, top), with anti-Runx2 antibody (B, bottom) or with anti-Cathepsin-K (C). Magnified insert are shown on the right. Arrows indicate Bone matrix, Runx2 or Cathepsin-K positive cells at B top or bottom and C panel respectively.

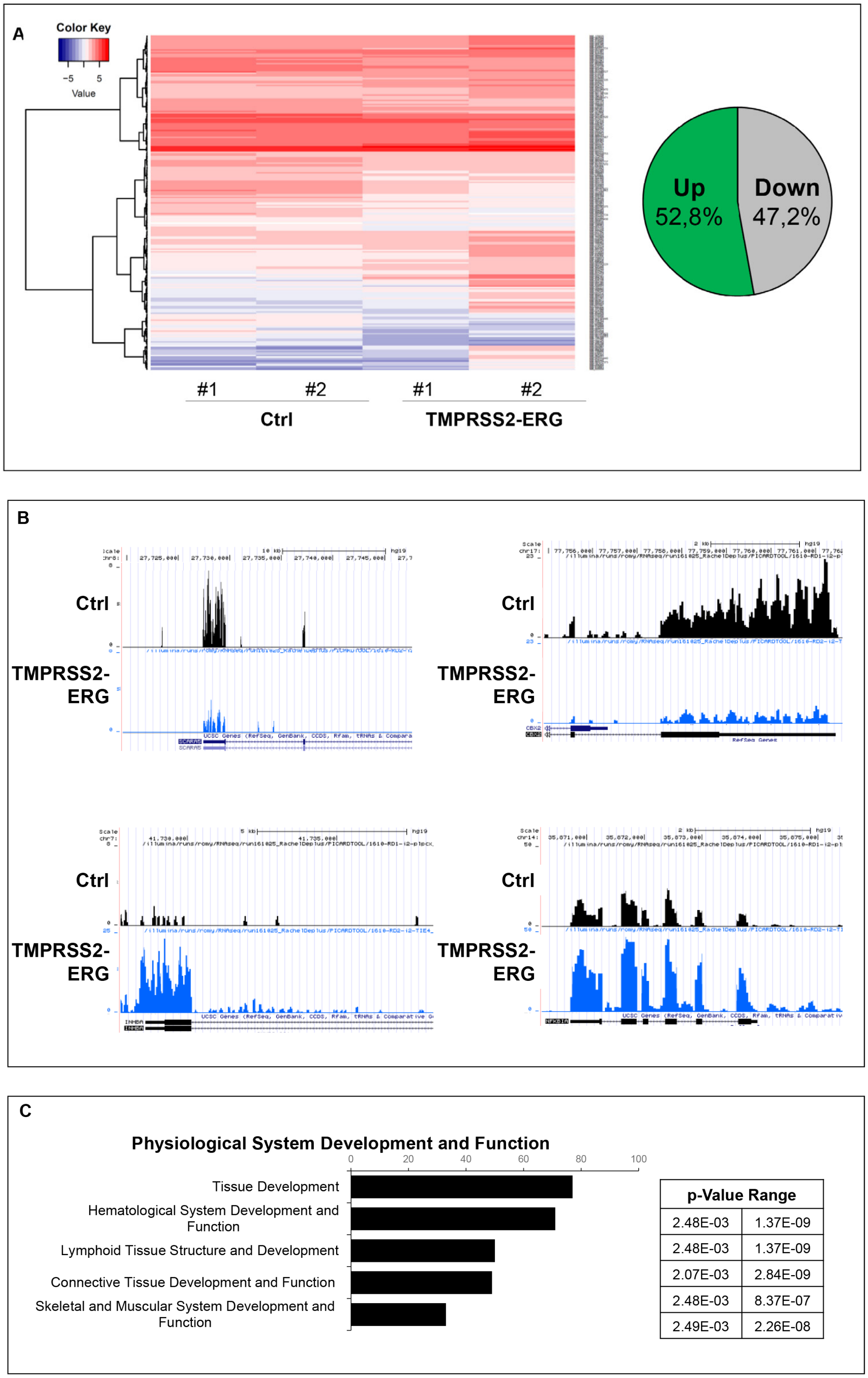

Supplementary Figure 7 (related to Figure 4): A. Heatmap showing differentially expressed genes between PC3M-luc Ctrl and TMPRSS2-ERG cells. A technical duplicate was analysed for each sample (#1 and #2). The heatmap scale shows the range of expression level, whereby positive (red) and negative (blue) values correspond, respectively, to a higher and a lower expression. **B.** Additional examples of RNA-Seq peaks. **C.** Ingenuity Pathway Analysis (IPA®) ("Physiological System Development and Function") of the differentially expressed genes. The x axis represents the number of molecules per categories. Range of p-value is indicated.

Supplementary Table 1: sequences of primers used for RT-qPCR

Name	Sequence 5'-3'	Application
**ERG-Fwd**	AACGAGCGCAGAGTTATCGT	RT-qPCR
**ERG-Rev**	GTGAGCCTCTGGAAGTCGTC	RT-qPCR
**CBX2-Fwd**	CCGAGTGCATCCTGAGCAA	RT-qPCR
**CBX2-Rev**	GCTCCCAGCTGTTATGTTTGG	RT-qPCR
**CREB3L1-Rev**	CTGGAGAATGCCAACAGGACC	RT-qPCR
**CREB3L1-Fwd**	AGAACAAAGCACAAGGCTGC	RT-qPCR
**GNG11-Fwd**	GGCCTTCAGTTGTTTCGGGA	RT-qPCR
**GNG11-Rev**	TTAGACACTTGTTGTCTCTGCAA	RT-qPCR
**SCARA5-Fwd**	ATGCGTGGGTTCAAAGGTGA	RT-qPCR
**SCARA5-Rev**	GACCTGAGCCATTCACCAGG	RT-qPCR
**SIGLC15-Fwd**	GGTTCTCCCGACAGGCTCATT	RT-qPCR
**SIGLC15-Rev**	GCACCTCTGTGTTGAGCAAGT	RT-qPCR
**SDHA-Fwd**	TGGGAACAAGAGGGCATCTG	RT-qPCR
**SDHA-Rev**	CCACCACTGCATCAAATTCATG	RT-qPCR
**HPRT-Fwd**	TGACACTGGCAAAACAATGCA	RT-qPCR
**HPRT-Rev**	GGTCCTTTTCACCAGCAAGCT	RT-qPCR
**GAPDH-Fwd**	GGGGAGGCGTGTGTGTC	RT-qPCR
**GAPDH-Rev**	ATGGTGTCTGAGCGATGTGG	RT-qPCR
**OSTERIX-Fwd**	AGAGCTAGGAGGACAGAGGC	RT-qPCR
**OSTERIX-Rev**	CTTGAGACAGCAGGGGACAG	RT-qPCR
**RUNX2-Fwd**	TGCTGGAGTGATGTGGTTTTC	RT-qPCR
**RUNX2-Rev**	TTTGCTATGAAGCCTGGCGA	RT-qPCR
**RANK-Fwd**	AGATGGCTTTCCCAGTGTGT	RT-qPCR
**RANK-Rev**	GCAACTATCTCCAGGCCTCA	RT-qPCR

Supplementary Table 2: Ingenuity pathway analysis "Top Disease and Bio Functions": List of molecules implied in cancer

See Supplementary File 1

Supplementary Table 3: Ingenuity pathway analysis "Molecular and Cellular Functions": List of molecules implied in Cell-To-Cell Signaling and Interaction

See Supplementary File 2

Supplementary Table 4: Ingenuity pathway analysis "Molecular and Cellular Functions": List of molecules implied in Cellular Movement

See Supplementary File 3

Supplementary Table 5: Ingenuity pathway analysis "Physiological System Development and Function": List of molecules implied in Connective Tissues Development and Function

See Supplementary File 4

Supplementary Table 6: List of differentially expressed genes between PC3M-luc Ctrl and TMPRSS2-ERG cells

See Supplementary File 5
